# Outcomes of Ab Interno 63 µm vs. 45 µm XEN^®^ Gel Stent in Open-Angle Glaucoma: A Five-Year Follow-Up Study

**DOI:** 10.3390/jcm15083028

**Published:** 2026-04-15

**Authors:** Thomas Jacobs, Marie-Isaline Billen Moulin-Romsée, Victor Raeymaeckers, Nawid Faizi, Nathan M. Kerr, Keith R. Martin, Jan Van Eijgen, Ingeborg Stalmans

**Affiliations:** 1 Research Group Ophthalmology, Department of Neurosciences, KU Leuven, 3000 Leuven, Belgium; 2 Department of Ophthalmology, University Hospitals UZ Leuven, 3000 Leuven, Belgium; 3 Centre for Eye Research Australia, East Melbourne, VIC 3002, Australia; 4 Royal Victorian Eye and Ear Hospital, East Melbourne, VIC 3002, Australia; 5 Ophthalmology, Department of Surgery, University of Melbourne, Melbourne, VIC 3010, Australia

**Keywords:** XEN Gel Stent, XEN45, XEN63, ab interno, minimally invasive bleb surgery, mibs, bleb-forming devices without a plate, intraocular surgery, glaucoma, open-angle glaucoma

## Abstract

**Background/Objectives**: To compare the five-year efficacy and safety of the 63 µm (XEN63) vs. 45 µm (XEN45) XEN^®^ Gel Stent in patients with open-angle glaucoma (OAG). **Methods**: This retrospective matched (1:1) cohort study included adults with OAG who underwent standalone ab interno implantation of the XEN63 or the XEN45 between 2014 and 2021 at a tertiary referral center in Belgium. The primary outcome was IOP at five years. The secondary outcomes included surgical success, topical medication use, postoperative hypotony, complications and interventions. **Results**: Thirty eyes of 30 patients (15 XEN63 and 15 XEN45) were analyzed. The baseline characteristics were comparable. At five years, the mean IOP did not differ between the XEN63 and the XEN45 (11.5 vs. 11.0 mmHg; *p* = 0.54). The XEN63 demonstrated higher complete success rates than the XEN45 for both the IOP < 18 mmHg (10 vs. four eyes; *p* = 0.016) and <15 mmHg criteria (10 vs. three eyes; *p* = 0.003). The topical medication use was low and comparable (0.6 vs. 0.9 medications; *p* = 0.57). The numerical (13 vs. five eyes; *p* = 0.008) and symptomatic (six vs. two eyes; *p* = 0.2) hypotony were more frequent after the XEN63 implantation. The two eyes with XEN63 and none with XEN45 experienced clinically significant hypotony. The needling procedures and secondary glaucoma surgeries were more frequent after the XEN45. **Conclusions**: The XEN63 implantation was associated with higher long-term success rates and also with a higher incidence of early postoperative hypotony. These findings indicate a trade-off between efficacy and safety and suggest that careful patient selection and postoperative management are essential when considering larger lumen subconjunctival drainage devices.

## 1. Introduction

Over the past two decades, numerous surgical modalities have been introduced into the clinical practice of glaucoma surgery [1]. Among these devices is the XEN^®^ Gel Stent (AbbVie Inc., North Chicago, IL, USA), a bleb-forming device without a plate, designed to divert aqueous humor from the anterior chamber towards the subconjunctival space [2,3]. This microstent is composed of glutaraldehyde-crosslinked porcine gelatine, measures 6 mm in length and is manufactured with an internal luminal diameter of 45 or 63 µm, which is called 45 µm XEN^®^ Gel Stent (XEN45) and 63 µm XEN^®^ Gel Stent (XEN63) [2].

Under laminar flow conditions, the Hagen–Poiseuille law predicts that volumetric flow increases with the radius (intraluminal diameter) to the fourth power [4]. Consequently, the larger lumen of the XEN63 is expected to confer a substantially lower flow resistance than the XEN45, with minor differences in luminal diameter that theoretically result in marked changes in the aqueous outflow.

The currently available evidence evaluating the clinical outcomes of the XEN63 remains limited. As highlighted in the recent systematic review by Traverso et al., much of the early literature on the XEN63 is based on precursor devices that were not commercially released [2]. To date, only a small number of studies have assessed the performance of the currently available XEN63 implants in clinical practice [5,6,7,8,9,10].

Among these, Fea et al. reported significant intraocular pressure (IOP) reductions with the XEN63 and a mean decrease of approximately 14.8 mmHg at three months [7]. In a subsequent real-world study with 18 months of follow-up, a sustained IOP reduction and a decrease in the number of topical glaucoma medications were observed, with a low incidence of (mostly mild) adverse events [8].

However, in clinical practice, the choice between the XEN63 and the XEN45 reflects a key trade-off: the larger lumen of the XEN63 may enable a greater IOP reduction but may also increase the risk of early postoperative hypotony. This balance between efficacy and safety remains insufficiently characterized, particularly in the long term. This study aims to be the first ever to juxtapose the five-year efficacy and safety profile of the XEN63 with that of the XEN45.

## 2. Materials and Methods

A retrospective single-center cohort study comparing patients with open-angle glaucoma who underwent a standalone ab interno XEN63 or XEN45 implantation. This study adhered to the tenets of the Declaration of Helsinki and received approval from the Ethics Committee of the University Hospitals Leuven (reference number: S71269), which was the sponsor of this research.

### 2.1. Participants

All the patients aged 18 or over who received a standalone ab interno XEN63 between 2014 and 2021 in the University Hospitals (UZ) Leuven with a diagnosis of primary open-angle glaucoma, normal tension glaucoma, pseudoexfoliative glaucoma or pigmentary glaucoma were included. The patients with any other form of glaucoma were excluded.

From the electronic medical records, the following parameters were extracted: (1) the baseline information, which included the demographic data, glaucoma diagnosis, ocular history, medication use, visual acuity, pre-operative IOP measures, visual field mean deviation, and central corneal thickness (CCT); (2) the pre-operative information, which included the surgical device, type of anesthesia and complications; and (3) the postoperative information, which included the IOP, topical medications, oral acetazolamide use, complications, surgical interventions and secondary glaucoma surgery.

An equal number of patients who underwent the XEN45 implantation in the same period were manually and blindly matched (1:1) retrospectively based on their glaucoma diagnosis, gender, age and pre-operative IOP. If the pre-operative IOP did not match exactly, then the patient with the same glaucoma diagnosis and gender and the closest pre-operative IOP was chosen. When multiple potential matches were available, the patient with the closest age, based on the exact birthdate, was selected. Only one eye per patient was included.

### 2.2. Surgical Technique and Postoperative Care

All procedures were performed by the Glaucoma Department of the University Hospitals (UZ) Leuven by one experienced glaucoma surgeon (IS). Both models were implanted using a standardized, standalone ab interno approach with a preloaded injector and without opening the conjunctiva. For the implantation of the XEN63, a 25G injector was utilized, whereas the XEN45 was implanted using a 27G injector. The surgical technique is identical for both devices and has been described elsewhere [11]. Regarding the antifibrotics, subconjunctival mitomycin C (MMC 0.01%) injections were administered immediately prior to the XEN placement and intracameral bevacizumab at the end of the procedure.

The postoperative management included topical antibiotic therapy for one month. The topical corticosteroids were prescribed for three months, with a gradual tapering during the final month.

### 2.3. Outcomes

The primary outcome of this study was the IOP at five years post-operation. The surgical success, which was a secondary outcome, was defined in two categories: (1) complete success, which required an IOP of < 18 or < 15 mmHg without any hypotony-related complications (i.e., shallowing of the AC, maculopathy, or choroidal swelling that require intervention), without the need for additional topical medications or laser trabeculoplasty, and in the absence of secondary glaucoma surgeries, surgical revision, or total loss of light perception, and (2) qualified success, which applied the same criteria but allowed the use of supplemental topical therapy or laser trabeculoplasty. Other secondary outcomes included the number of topical medications, postoperative hypotony (IOP < 6 mmHg, cfr. infra), complications, interventions, and secondary glaucoma surgery within the five-year follow-up period.

The postoperative hypotony was classified into the following categories: (1) numeric hypotony has an IOP of <6 mmHg; (2) symptomatic hypotony has an IOP of <6 mmHg with hypotony-related complications (i.e., shallowing of the AC, maculopathy, or choroidal swelling that requires intervention); and (3) clinically significant hypotony has an IOP of <6 mmHg with hypotony-related complications (cfr. supra) that require intervention.

### 2.4. Statistical Analysis

All the statistical analyses were performed using R (version 4.4.2; R Foundation for Statistical Computing, Vienna, Austria). A *p*-value of 0.05 was considered the threshold for statistical significance. No formal adjustments for multiple testing were performed.

The time-to-failure analyses for complete and qualified success were performed using Kaplan–Meier survival methods. The time zero was defined as the date of the device implantation. Failure was defined as the first occurrence of a loss of the complete or qualified success criteria. The Kaplan–Meier curves were constructed separately for the XEN63 and the XEN45, and the cumulative probabilities of complete and qualified success, with 95% confidence intervals, were estimated at 1-, 2-, 3-, 4-, and 5-year intervals and separately compared with a pointwise KM Z-test. The survival distributions between the devices were compared using the log-rank test.

The IOP and number of topical medications were summarized using the mean and standard deviation of each postoperative time point. The distributional assumptions were assessed using the Shapiro–Wilk test. The between-group comparisons of the IOP and medication use at each interval were performed using independent samples tests (a *t*-test or a Mann–Whitney U test, as appropriate). The incidences of postoperative hypotony, complications, additional interventions, and secondary glaucoma surgery were reported as proportions. The comparisons between devices for categorical outcomes were conducted using Fisher’s exact test.

The missing IOP and topical medication values across postoperative time points were addressed using multiple imputations with chained equations. All the longitudinal IOP and topical medication variables and device types were included as predictors. Continuous values were imputed using predictive mean matching, and five imputed datasets were generated with 20 iterations each. The summary statistics were computed within each dataset, and the pooled mean value estimates with 95% confidence intervals were derived using Rubin’s rules.

The longitudinal postoperative data were variably available across the follow-up visits in both the treatment groups. In the XEN63 cohort, the number of complete IOPs and medication records was 9 in year 1, 10 in year 2, 13 in year 3, 12 in year 4, and 10 in year 5. In the XEN45 cohort, the available datapoints were 12 in year 1, 11 in year 2, 13 in year 3, 12 in year 4, and 11 in year 5. The missingness resulted primarily from a failure to follow up or death.

## 3. Results

### 3.1. Study Population

The baseline demographics and ocular characteristics showed no statistical differences between the XEN63 and the XEN45 (see Table 1). Nevertheless, two patients (13%) in the XEN63 group were taking oral carbonic anhydrase inhibitors prior to the surgery, compared to no patients in the XEN45 group (*p* = 0.48).

### 3.2. Efficacy

#### 3.2.1. Intraocular Pressure

**Figure 1:**
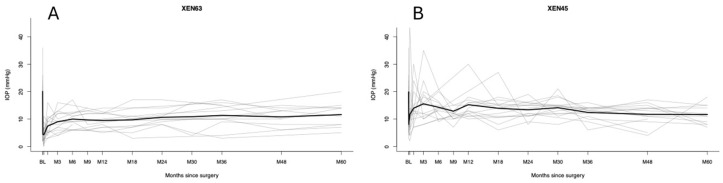
A spaghetti plot with the observed individual and mean intraocular pressure (IOP) trajectories following (**A**) the XEN63 implantation and (**B**) the XEN45 implantation. The thin gray lines represent the individual patient IOP values across the follow-up visits, and the thick black line depicts the mean IOP at each postoperative time point.

**Figure 2:**
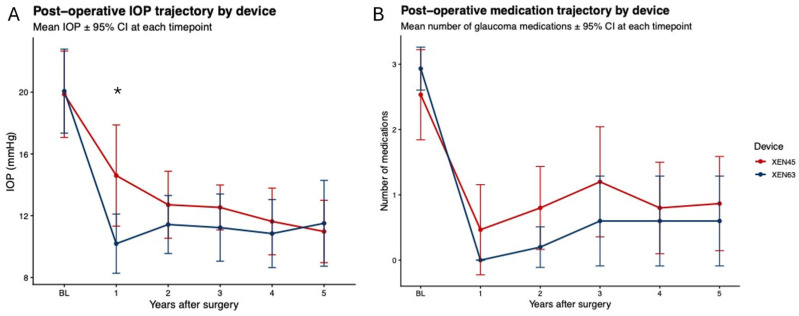
The imputed mean of (**A**) the intraocular pressure (IOP) and (**B**) the number of topical medications over time following the implantation of the XEN63 (blue) and the XEN45 (red) devices. The values represent the group means with 95% confidence intervals at each postoperative year. The statistically significant differences are indicated with asterisks (* *p* < 0.05).

In year 1, the XEN63 achieved a lower IOP than the XEN45 (10.2 mmHg vs. 14.6 mmHg; *p* = 0.009) (see Table 2 and Figure 1 and Figure 2A). From year 2 to year 5, the IOP remained consistently lower in the XEN63, although the differences were not statistically significant: year 2 (11.4 vs. 12.7 mmHg; *p* = 0.26), year 3 (11.2 vs. 12.5 mmHg; *p* = 0.27), year 4 (10.8 vs. 11.6 mmHg; *p* = 0.32), and year 5 (11.5 vs. 11.0 mmHg; *p* = 0.54).

#### 3.2.2. Topical Medications

In year 1, the medication use dropped to 0 in the XEN63 group (95% CI: 0-0), compared with a medication use of 0.5 in the XEN45 group (95% CI: 0–1.2; *p* = 0.17) (see Table 2 and Figure 2B). From Year 2 onward, both groups required few medications, with no statistically significant differences observed: year 2 (0.2 vs. 0.8; *p* = 0.08), year 3 (0.6 vs. 1.2; *p* = 0.25), year 4 (0.6 vs. 0.8; *p* = 0.67), and year 5 (0.6 vs. 0.9; *p* = 0.57).

#### 3.2.3. Surgical Success

**Figure 3:**
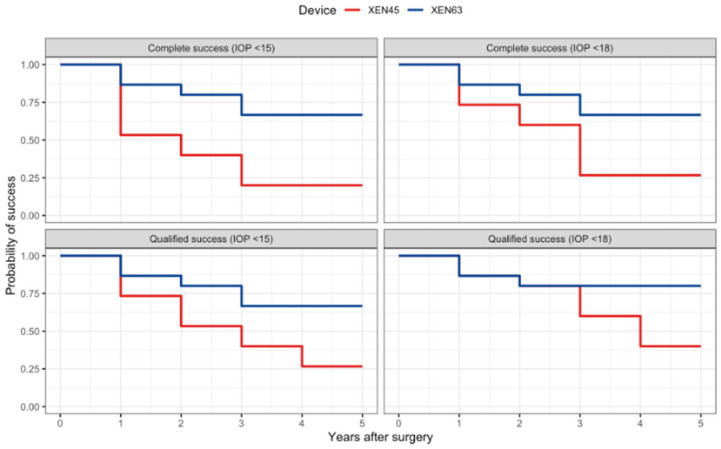
The Kaplan–Meier survival curves showing the complete and qualified success rates over five years for the XEN63 and the XEN45. The Kaplan–Meier estimates for the surgical success of the XEN63 (blue) and the XEN45 (red) are presented for the four success definitions: complete success with a target intraocular pressure (IOP) of < 15 mmHg (**top left**), complete success with a target IOP of < 18 mmHg (**top right**), qualified success with a target IOP of < 15 mmHg (**bottom left**), and qualified success with a target IOP of < 18 mmHg (**bottom right**).

Across the five-year follow-up period, the XEN63 demonstrated higher complete success rates than the XEN45 under the IOP < 18 mmHg criterion (*p* = 0.04) and the <15 mmHg criterion (*p* = 0.008) (see Table 3 and Figure 3). In year five, the XEN63 and the XEN45 achieved complete success in 67% and 27% of the patients (10 vs. four eyes; *p* = 0.016) using the <18 mmHg criterion, respectively, and in 67% and 20% of the patients (10 vs. three eyes; *p* = 0.003) using the <15 mmHg criterion.

Across the five-year follow-up period, a higher qualified success rate for the XEN63 was observed using the <15 mmHg criterion (*p* = 0.04) but not using the <18 mmHg criterion (*p* = 0.05) (see Table 3 and Figure 3). For qualified success in year five, the XEN63 and the XEN45 achieved success in 80% and 40% of patients (12 vs. six eyes; *p* = 0.014 using the <18 mmHg criterion), respectively, and in 67% and 27% of the patients (10 vs. four eyes; *p* = 0.016) using the <15 mmHg criterion.

### 3.3. Safety

**Figure 4:**
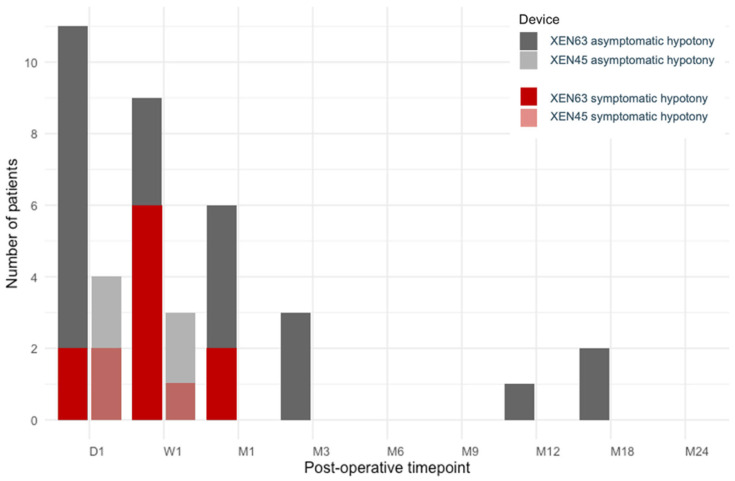
The number of patients with postoperative hypotony (an intraocular pressure of < 6 mmHg) at early postoperative time points. The darker shades (**left**) depict the XEN63, and the lighter shades depict the XEN45 (**right**). The gray bars represent asymptomatic hypotony, and the red bars represent symptomatic hypotony.

The majority of the postoperative hypotony for both devices occurred in the early postoperative phase (see Figure 4). The numerical hypotony was more frequent in the XEN63 compared to the XEN45 (13 vs. five; *p* = 0.008), while both devices showed a gradual decline in incidence over time. The symptomatic hypotony occurred non-significantly more in the XEN63 compared to the XEN45 (six vs. two, *p* = 0.2). In the XEN63 group, the complications included choroidal detachment in four patients (27%), macular folding in one patient (7%), and choroidal bleeding in one patient (7%). Of these six patients, two had clinically significant hypotony, while the remaining four experienced transient findings that spontaneously resolved with conservative management within four weeks. In comparison, two patients (13%) in the XEN45 group developed choroidal detachment, and neither had clinically significant hypotony.

The postoperative interventions were less frequently required following the XEN63 compared to the XEN45 implantation. The needling procedures were performed in one eye (7%) in the XEN63 group vs. seven eyes (47%) in the XEN45 group (*p* = 0.04). Secondary glaucoma surgery was also less common after the XEN63 implantation, occurring in one eye (7%) compared to six eyes (40%) in the XEN45 group (*p* = 0.08). Other interventions, including XEN revision, suturing for positive Seidel, anterior chamber repair, anterior chamber flushing, and laser trabeculoplasty, were infrequent and comparable between groups (see Table 4).

## 4. Discussion

Although a great body of evidence supports the effectiveness of the XEN45, the body of literature evaluating the XEN63 remains limited [2]. The XEN63 has been shown to be effective in short-term IOP lowering and topical medication in single-arm studies reduction [5,7,8,12,13]. In all but one study, an ab interno closed conjunctiva approach was used [5,7,8]; the study by Bertolani et al. used an ab externo, open conjunctiva approach [12]. Two prospective non-comparative studies have also reported a sustained IOP reduction in the intermediate term, up to three years postoperatively [6,10].

Three prior comparative studies have evaluated the XEN63 and the XEN45 up to one year postoperatively, collectively showing higher success rates (i.e., lower IOPs and lower topical medications), with similar or more postoperative interventions and adverse events for the XEN63 [14,15,16].

One study, with follow-up results up to three years, demonstrated an early and intermediate postoperative IOP that was higher for the XEN63 compared to the XEN45 [9]. These results by Fernández-García et al. are counterintuitive with respect to the Hagen–Poiseuille equation [4]. It can be hypothesized that the discrepancy may be attributable to the lower surgical experience with the XEN63 compared to the XEN45 at the time of implantation in this study [9].

To the best of our knowledge, the present study provides the first long-term comparison between the two types of stents. Our findings indicate that the XEN63 provides higher long-term surgical success compared with the XEN45, while maintaining an acceptable safety profile. However, the XEN63 is associated with a higher incidence of early postoperative hypotony and related complications. Two patients in the XEN63 group were using oral carbonic anhydrase inhibitors, which may have increased their risk for early postoperative hypotony [17]. Another explanation for this high prevalence can be found in the use of the 25G injector for the XEN63, which has been replaced by a new 27G version, thereby reducing the risk for early postoperative peritubular flow and hypotony [7]. Nevertheless, fewer needlings had to be performed in the XEN63 group, indicating a potential trade-off between the reduced downstream interventions at the cost of higher rates of early postoperative hypotony. A similar concept has been described in a PRESERFLO™ MicroShunt (Santen SA, Osaka, Japan) study by Steiner et al., where the use of a larger-diameter needle tract (27G vs. 25G) was associated with improved early postoperative outcomes at the cost of more hypotony-related complications [18]. When an ab externo open conjunctiva approach is selected for the XEN63 implantation, the placement of an intraluminal stent may help mitigate this early postoperative hypotony. This strategy has been successfully adopted in the PRESERFLO™ MicroShunt implantation with encouraging results [17,19,20].

The observed differences in efficacy and safety profiles between the XEN63 and the XEN45 suggest that both device sizes may have a complementary role in routine clinical practice. Our findings support a more individualized, patient-tailored approach to surgical decision-making, in which the device selection is guided by the balance between the desired IOP lowering and the patient’s risk profile. In particular, the lower rate of hypotony observed with the XEN45 may be advantageous in patients at an increased risk of hypotony-related complications, such as those with high myopia or other predisposing factors. Conversely, the higher long-term surgical success observed with the XEN63 may suggest its favor in patients requiring a more pronounced or sustained pressure-lowering. However, these considerations remain hypothesis-generating, and further prospective studies are warranted to establish more definitive guidance on device selection.

The retrospective nature of this study is inherently subject to information and selection bias, which represents one of the limitations of this study. Matching on the pre-operative IOP, glaucoma diagnosis, age, and sex likely captures the dominant determinants of the early surgical outcomes; however, it does not encompass all the aspects of underlying disease severity. Importantly, the small sample size represents a key limitation. It limits the statistical power, complicates the correct interpretation of multiple imputation, and may also limit the generalizability of the findings. The longitudinal IOP and medication data were analyzed using multiple imputations by chained equations, assuming data were missing at random, as a failure to follow up was primarily driven by logistical factors rather than clinical outcomes. By performing multiple comparisons across time points and using multiple imputations, these analyses were exploratory and intended to support trends rather than establish definitive effects. Nonetheless, the long-term trends that were observed align with the previously reported short-term outcomes [9,14,15,16]. In addition, the follow-up visual field mean deviation data were scarce, precluding the robust assessment of functional progression over time. Lastly, as all the procedures were performed by a single experienced surgeon, the results may be a reflection of surgeon-specific technique and expertise, which may limit the external generalizability, although it ensures procedural consistency between both devices.

While exploratory, the observed trends may help generate hypotheses for future studies exploring device design and flow-modulation strategies that are aimed at improving surgical predictability.

## 5. Conclusions

Despite similar long-term IOP levels, the XEN63 was associated with higher rates of surgical success over time, alongside a substantially higher incidence of early postoperative hypotony and related complications. In contrast, the XEN45 implantation was followed by a higher need for postoperative interventions and secondary glaucoma surgery.

These findings suggest that an increased stent lumen diameter may influence the probability of achieving the medication-free target IOP at the cost of an increased early hypotony risk. Given the retrospective design and limited sample size, these results should be interpreted cautiously. Further prospective studies are required to clarify the risk-benefit balance of larger-lumen subconjunctival drainage devices and to identify the strategies that may reduce hypotony while preserving long-term efficacy.

## Figures and Tables

**Table 1:** The baseline characteristics.

		XEN63	XEN45	*p*-Value
Eyes, n		15	15	
Sex, n (%)	Female	6 (40)	6 (40)	1
	Male	9 (60)	9 (60)	
Diagnosis, n (%)	POAG	11 (73)	11 (73)	1
	PEX	3 (20)	3 (20)	
	PG	1 (7)	1 (7)	
Severity (Hodapp classification), n (%)	Mild	4 (27)	5 (33)	0.81
	Moderate	5 (33)	3 (20)	
	Severe	6 (40)	7 (47)	
Lens status, n (%)	Phakic	6 (40)	8 (53)	0.71
	Pseudo-phakic	9 (60)	7 (47)	
Topology, n (%)	RE	11 (73)	10 (67)	1
	LE	4 (27)	5 (33)	
Taking oral CAI, n (%)	Yes	2 (13)	0 (0)	0.48
	No	13 (87)	15 (100)	
Age (years), mean ± SD		69.3 ± 11.6	66.7 ± 10.2	0.53
Number of topical meds, mean ± SD		2.9 ± 0.6	2.5 ± 1.3	0.275
IOP (mmHg), mean ± SD		20.1 ± 5.4	19.9 ± 5.5	0.92
Max IOP (mmHg), mean ± SD		27.4 ± 10.3	32.4 ± 9.5	0.23
CCT (µm), mean ± SD		551.6 ± 43.3	534.7 ± 44.6	0.32
BCVA (Snellen), mean ± SD		0.7 ± 0.2	0.73 ± 0.2	0.9
Previous Glaucoma Surgery, mean ± SD		0.3 ± 0.6	0.2 ± 0.4	0.49

BCVA = best corrected visual acuity, CAI = carbonic anhydrase inhibitors, CCT = central corneal thickness, IOP = intraocular pressure, LE = left eye, PG = pigmentary glaucoma, PEX = pseudoexfoliative glaucoma, and POAG = primary open-angle glaucoma, RE = right eye.

**Table 2:** The intraocular pressure and topical medication trends. The statistically significant results are indicated with asterisks (* *p* < 0.05).

	Intraocular Pressure (mmHg)					Number of Medications				
	XEN63		XEN45		*p*-Value	XEN63		XEN45		*p*-Value
	Mean	CI Lower 95–CI Upper 95	Mean	CI Lower 95–CI Upper 95		Mean	CI Lower 95–CI Upper 95	Mean	CI Lower 95–CI Upper 95	
Baseline	20.1	17.4–22.8	19.9	17.1–22.7	0.921	2.9	2.6–3.3	2.5	1.8–3.2	0.275
Year 1	10.2	8.3–12.1	14.6	11.3–17.9	0.009 *	0	0–0	0.5	−0.2–1.2	0.169
Year 2	11.4	9.6–13.3	12.7	10.5–14.9	0.256	0.2	−0.1–0.5	0.8	0.2–1.4	0.083
Year 3	11.2	9.1–13.4	12.5	11.1–14	0.273	0.6	−0.1–1.3	1.2	0.4–2	0.247
Year 4	10.8	8.6–13	11.6	9.5–13.8	0.323	0.6	−0.1–1.3	0.8	0.1–1.5	0.666
Year 5	11.5	8.7–14.3	11	9–13	0.535	0.6	−0.1–1.3	0.9	0.1–1.6	0.571

**Table 3:** The comparative survival analysis between the XEN63 and the XEN45 across the four success criteria: complete success at an intraocular pressure (IOP) of < 15 mmHg, complete success at an IOP of < 18 mmHg, qualified success at an IOP of < 15 mmHg, and qualified success at an IOP of < 18 mmHg. For each definition, both the overall difference across the five-year follow-up and the pointwise differences at each postoperative year are shown. The statistically significant results are indicated with asterisks (* *p* < 0.05; ** *p* < 0.01).

		Success XEN63 n (%)	Success XEN45 n (%)	*p*-Value
Complete success (IOP < 18 mmHg)	Overall			0.040 *
	Year 1	13 (87)	11 (73)	0.352
	Year 2	12 (80)	9 (60)	0.221
	Year 3	10 (67)	4 (27)	0.017 *
	Year 4	10 (67)	4 (27)	0.017 *
	Year 5	10 (67)	4 (27)	0.017 *
Complete success (IOP < 15 mmHg)	Overall			0.008 **
	Year 1	13 (87)	8 (53)	0.032 *
	Year 2	12 (80)	6 (40)	0.014 *
	Year 3	10 (67)	3 (20)	0.003 **
	Year 4	10 (67)	3 (20)	0.003 **
	Year 5	10 (67)	3 (20)	0.003 **
Qualified success (IOP < 18 mmHg)	Overall			0.051
	Year 1	13 (87)	13 (87)	1
	Year 2	12 (80)	12 (80)	1
	Year 3	12 (80)	9 (60)	0.221
	Year 4	12 (80)	6 (40)	0.014 *
	Year 5	12 (80)	6 (40)	0.014 *
Qualified success (IOP < 15 mmHg)	Overall			0.037 *
	Year 1	13 (87)	11 (73)	0.352
	Year 2	12 (80)	8 (53)	0.106
	Year 3	10 (67)	6 (40)	0.128
	Year 4	10 (67)	4 (27)	0.017 *
	Year 5	10 (67)	4 (27)	0.017 *

**Table 4:** The postoperative interventions. The statistically significant results are indicated with asterisks (* *p* < 0.05).

	XEN63	XEN45	*p*-Value
Needling	1 (7%)	7 (47%)	0.04 *
XEN revision	1 (7%)	0 (0%)	1
Suturing (for positive Seidel)	1 (7%)	0 (0%)	1
AC repair	1 (7%)	0 (0%)	1
AC flushing	1 (7%)	0 (0%)	1
SLT/ALT	0 (0%)	2 (13%)	1
Secondary glaucoma surgery	1 (7%)	6 (40%)	0.08

AC = anterior chamber, ALT = argon laser trabeculoplasty, and SLT = selective laser trabeculoplasty.

## Data Availability

The data underlying this study are not publicly available due to ethical restrictions but can be made available from the corresponding author upon reasonable request.

## Abbreviations

The following abbreviations are used in this manuscript:

AC	Anterior Chamber
ALT	Argon Laser Trabeculoplasty
BCVA	Best Corrected Visual Acuity
CAI	Carbonic Anhydrase Inhibitors
CCT	Central Corneal Thickness
IOP	Intraocular Pressure
KM	Kaplan–Meier
LE	Left Eye
MIBS	Minimally Invasive Bleb Surgery
MMC	Mitomycin C
NTG	Normal Tension Glaucoma
OAG	Open-Angle Glaucoma
PEX	Pseudoexfoliative Glaucoma
PG	Pigmentary Glaucoma
POAG	Primary Open-Angle Glaucoma
RE	Right Eye
SD	Standard Deviation
SLT	Selective Laser Trabeculoplasty
XEN45	45 µm XEN^®^ Gel Stent
XEN63	63 µm XEN^®^ Gel Stent
